# Amelioration of Renal Inflammation, Endoplasmic Reticulum Stress and Apoptosis Underlies the Protective Effect of Low Dosage of Atorvastatin in Gentamicin-Induced Nephrotoxicity

**DOI:** 10.1371/journal.pone.0164528

**Published:** 2016-10-11

**Authors:** Krit Jaikumkao, Anchalee Pongchaidecha, La-ongdao Thongnak, Keerati Wanchai, Phatchawan Arjinajarn, Varanuj Chatsudthipong, Nipon Chattipakorn, Anusorn Lungkaphin

**Affiliations:** 1 Department of Physiology, Faculty of Medicine, Chiang Mai University, Chiang Mai, Thailand; 2 School of Medicine, Mae Fah Luang University, Chiang Rai, Thailand; 3 Department of Biology, Faculty of Science, Chiang Mai University, Chiang Mai, Thailand; 4 Department of Physiology, Faculty of Science, Mahidol University, Bangkok, Thailand; 5 Cardiac Electrophysiology Research and Training Center, Faculty of Medicine, Chiang Mai University, Chiang Mai, Thailand; Massachusetts Eye & Ear Infirmary, Harvard Medical School, UNITED STATES

## Abstract

Gentamicin is a commonly used aminoglycoside antibiotic. However, its therapeutic use is limited by its nephrotoxicity. The mechanisms of gentamicin-induced nephrotoxicity are principally from renal inflammation and oxidative stress. Since atorvastatin, 3-hydroxy-3-methylglutaryl coenzyme A reductase inhibitors, exerts lipid-lowering effects, antioxidant, anti-inflammatory as well as anti-apoptotic effects, this study aimed to investigate the protective effects of atorvastatin against gentamicin-induced nephrotoxicity. Male Sprague Dawley rats were used and nephrotoxicity was induced by intraperitoneal injection of gentamicin, 100 mg/kg/day, for 15 days. Atorvastatin, 10 mg/kg/day, was administered by orally gavage 30 min before gentamicin injection on day 1 to 15 (pretreatment) or on day 10 to15 (delayed treatment). For only atorvastatin treatment group, it was given on day 1 to 15. At the end of the experiment, kidney weight, blood urea nitrogen and serum creatinine as well as renal inflammation (NF-κB, TNFαR1, IL-6 and iNOS), renal fibrosis (TGFβ1), ER stress (calpain, GRP78, CHOP, and caspase 12) and apoptotic markers (cleaved caspase-3, Bax, and Bcl-2) as well as TUNEL assay were determined. Gentamicin-induced nephrotoxicity was confirmed by marked elevations in serum urea and creatinine, kidney hypertrophy, renal inflammation, fibrosis, ER stress and apoptosis and attenuation of creatinine clearance. Atorvastatin pre and delayed treatment significantly improved renal function and decreased renal NF-κB, TNFαR1, IL-6, iNOS and TGFβ1 expressions. They also attenuated calpain, GRP78, CHOP, caspase 12, Bax, and increased Bcl-2 expressions in gentamicin-treated rat. These results indicate that atorvastatin treatment could attenuate gentamicin-induced nephrotoxicity in rats, substantiated by the reduction of inflammation, ER stress and apoptosis. The effect of atorvastatin in protecting from renal damage induced by gentamicin seems to be more effective when it beginning given along with gentamicin or pretreatment.

## Introduction

Nephrotoxicity is an adverse effect of gentamicin treatment, despite its positive therapeutic activity against both gram-positive and gram negative bacteria [1–3]. Gentamicin-induced nephrotoxicity occurs by its selective accumulation in the renal proximal convoluted tubules leading to glomerular atrophy, tubular necrosis, tubular fibrosis, and inflammation. The mechanisms of gentamicin-induced nephrotoxicity are principally from renal inflammatory cascades and elevated renal oxidative stress [4]. Renal inflammation is demonstrated by an infiltration of inflammatory cells such as monocytes and macrophages and the subsequent release of proinflammatory cytokines and activation of NF-κB in response to oxidative stress [1,3]. In addition, apoptosis and necrosis of renal tubular epithelial cells [5–7] and activation of renal matrix metalloproteinase [8] are also found in case of gentamicin-induced nephrotoxicity. The accumulation of gentamicin in the endoplasmic reticulum (ER) may induce endoplasmic reticulum (ER) stress which activates the unfolded protein response (UPR) and cell cycle arrest [9]. Under conditions of UPR overload, the cell undergoes apoptosis, which is mediated by the classical route of calpain and caspase 12 [10]. Moreover, when unfolded proteins accumulate, the ER chaperone immunoglobulin heavy-chain-binding protein (BiP; also known as GRP78) expression is increased and dissociates from the ER receptors, leading to their activation and triggering the ER stress response [11]. The transcription factor CCAAT-enhancer-binding protein homologous protein (CHOP) expression is also increased in response to ER stress and plays an important role in the induction of apoptosis [12,13]. Accordingly, the use of several agents with anti-oxidant, anti-inflammation and anti-apoptosis activities and ER stress inhibition or an agent that posses multiple mechanisms of action may successfully prevent or ameliorate gentamicin-induced nephrotoxicity.

Numbers of clinical and experimental evidence demonstrated that the pharmacological effects of statins include not only lowering the levels of cholesterol but also the exertion of a variety of pleiotropic, such as inhibition of inflammatory response, improvement of endothelial function, antioxidant, antithrombotic and anti-apoptotic effects [14]. In patient with hypercholesterolemia, statins were instrumental in reducing the progression of atherosclerosis by inhibiting of monocyte activation, enhancing metalloprotease synthesis in the vessel walls and the production of pro-inflammatory cytokines interleukin (IL)-6, tumor necrosis factors (TNFα) and IL-1β [15,16]. Statins also suppressed acute and chronic inflammation by inhibiting edema formation, leukocyte-endothelial adhesion, production of inflammatory cytokines and transcription factors [17–19]. It has also been reported that atorvastatin prevents the toxic effects of gentamicin in the kidney via the inhibition of MAPK and NF-κB signaling pathways and iNOS expression [20]. However, details of the pleiotropic effects of statins on nephrotoxicity have not been clearly demonstrated.

This study was designed to investigate the protective effects of atorvastatin against gentamicin-induced toxicity in rat kidneys. We hypothesized that atorvastatin improves renal function by ameliorating an inflammation and ER stress related apoptosis pathways in gentamicin-induced nephrotoxicity.

## Materials and Methods

### Animal preparation and treatment

The 30 male Sprague-Dawley rats (250–300 g), 10–12 weeks of age, used in this study were obtained from the National Laboratory Animal Center, Mahidol University, Salaya, Thailand. The animal facilities and all protocols were approved by the Laboratory Animal Care and Use Committees at the Faculty of Medicine, Chiang Mai University, Chiang Mai, Thailand (Permit Number: 06/2559). All experimental rats were housed in a room maintained at 25 ± 1°C on a 12 h light/dark cycle and fed on a normal laboratory diet and water ad libitum. Only male rats were used to get rid of the fluctuation of sex hormone during menstrual cycle in female rats which might affect our results. It has been reported that gentamicin could induce the sensitivity of renal toxicity in male rats more than female rats [21,22].

Thirty rats were randomly divided into five groups (six rats per group). (1) the vehicle control (C) group received normal saline by gavage; (2) the gentamicin (G) group. The rat was injected intraperitoneally (i.p.) with 100 mg/kg/day of gentamicin (The Govt. Pharm.Org, Thailand) with the volume of 700–800 μl for 15 days; (3) the atorvastatin group, Ator (Lek Pharmaceuticals d.d, Slovenia) dissolved in 500 μl of 0.9% normal saline solution at dose of 10 mg/kg/day was administered by gavage feeding once a day on day 1 to 15; (4) the atorvastatin pretreatment (Pre) group, Ator was administered by gavage 30 min before gentamicin treatment for 15 days; and (5) atorvastatin delayed treatment (Delayed) group, gentamicin was injected every day for 15 days and Ator was administered on days 10 to 15 by gavage 30 min before the gentamicin treatment. Gentamicin was injected at the same period of time, 8.00 to 9.00 am, in all groups of experiment. Atorvastatin treatments were also given at 8:00 to 9:00 am throughout the experiment. The chosen gentamicin dose was based on that given by intraperitoneal administration in previous studies that showed the drug induced nephrotoxicity in rat models [23–25]. Atorvastatin used in this study was chosen from the dose that given by oral gavage in previous studies that showed nephroprotection as well as oxidative stress improvement [18–20] and from preliminary study. Thus, we selected atorvastatin in the minimal effective therapeutic dose 10 mg/kg/day as used in the clinical to evaluate the beneficial effects of this drug to protect the kidney from gentamicin-induced toxicity. The animals were monitored before and after receiving treatments in the morning and again in the evening. Blood samples were collected from tail vein by cutting the tail tip under isoflurane inhalation. After the last injected dose of gentamicin, each rat was kept individually in metabolic cage for 24 h urine collection. Urine was centrifuged at 1000 rpm for 10 min, to remove cells and debris and stored at -20°C until investigation. At the end of study, the animals were deeply anesthetized by sodium pentobarbital injection intraperitoneally at the dose of 100 mg/kg and blood and kidney tissue samples were collected for subsequent experiments. Following intraperitoneal injection (single dose) of pentobarbital, the isoflurane inhalation was used to maintain anesthesia of the animal throughout surgical protocols. The animals were observed throughout the experiment. The rats that have the severity symptoms such as lack of appetite, inanimate and severe diarrhea were killed before the endpoint of experiment by sodium pentobarbital injection intraperitoneally at the dose of 100 mg/kg and verify that an animal is dead before disposing of the carcass, by making sure there is no respiratory movement for at least 3 minutes. If the animal is deeply unconscious but respirations have not ceased, the inhalation of isoflurane is followed for additional security until respirations have stopped.

### Determination of renal function

To assess renal function, serum and urine creatinine were measured using an automatic biochemical analyzer at the Clinical Laboratory, Maharaj Nakhon Chiang Mai Hospital, Chiang Mai, Thailand. Relative kidney weight was calculated according to the formula: kidney weight/total bodyweight. The creatinine clearance (C_cr_) reflected to glomerular filtration rate (GFR) was calculated using the following equation:

$${\text{C}}_{\text{cr}}\left(\text{ml}/\text{min}\right)\;=\;\left(\text{urine creatinine x urine flow rate}\right)\;/\text{ serum creatinine}$$

### Tissue preparation and Western blot analysis

The renal cortical tissues were used to carry out a Western blot analysis. The renal cortex was gently cut from the outer part of the kidney, sections extending down for approximately 3–4 mm were cut using a microtome. Each cellular compartment, whole cell lysate, membrane and cytosolic fractions were prepared from renal cortical tissues using differential centrifugation technique. Briefly, renal cortical slices were homogenized in Mammalian cell Lytic buffer (Sigma, St. Louis, MO, USA) with a protease cocktail inhibitor (Roche Diagnostics, Indianapolis, IN, USA). Homogenates were centrifuged at 5,000 × g for 15 min at 4°C. Some of the supernatants were collected as whole cell lysate, and the remaining portion was centrifuged at 100,000 × g for 2 h at 4°C to obtain the membrane (pellet) fraction. The 5,000 ×g pellet was re-suspended and centrifuged at 10,000 × g 4°C for 10 min. The supernatant fraction from the centrifugation was designated as the nuclear fraction.

Whole cell lysate, membrane and nuclear fractions from the renal cortex were subjected to 10% SDS-polyacrylamide gel electrophoresis (SDS-PAGE), and subsequently transferred to a polyvinylidene fluoride (PVDF) membrane (Millipore, Billerica, MA, USA). The membranes were then blocked with 5% nonfat dry milk in Tris-buffered saline (TBS) containing 0.1% tween-20 (TBST) solution for 1 h at room temperature and subsequently probed with primary antibodies overnight at 4°C. The protein expressions of Bax (Millipore, Billerica, MA, USA), Bcl-2 (Cell Signaling Technology, Danvers, MA, USA), cleaved caspase-3 (Millipore, Billerica, MA, USA), NF-κB (Millipore, Billerica, MA, USA), iNOS (Santa Cruz Biotechnology, Santa Cruz, CA, USA), TNFα receptor1 orTNFαR1, IL-6 (Santa Cruz Biotechnology, Santa Cruz, CA, USA), TGFβ1 (Cell Signaling Technology, Danvers, MA, USA), calpain, GRP78, CHOP (Cell Signaling Technology, Danvers, MA, USA) and caspase 12 (Millipore, Billerica, MA, USA) were determined using western blot. Lamin b1 (Cell Signaling Technology, Danvers, MA, USA) or Na^+^-K^+^ ATPase (Millipore, Billerica, MA, USA) was used as a marker for the nuclear and membrane fraction, respectively. The β-actin (Millipore, Billerica, MA, USA) was used as a loading control for all samples. The membranes were washed three times with TBST and incubated with horseradish peroxidase (HRP)-conjugated goat anti-rabbit or anti-mouse secondary antibodies (Amersham, Arlington Heights, IL, USA) at room temperature for one hour and developed with ECL enhanced chemiluminescence agent (GE Healthcare, Buckinghamshire, UK). Each membrane was stripped and re-probed with mouse anti-β-actin antibody or another antibody for further detection of protein expression. The western blot film images were scanned and were analyzed using Image J (NIH image) analysis software.

### Determination of renal apoptosis by TUNEL assay

Apoptosis in renal tissues was identified by a Terminal deoxynucleotidyl transferase dUTP nick end labeling (TUNEL assay) with tissue paraffin blocks using TdT-FragEL^™^ DNA fragmentation detection kit (Millipore, Billerica, MA, USA) according to the manufacturer's instruction. Tissue sections were treated with proteinase K for 20 min. After TBS washing, the sections were incubated with DNase I for 20 min. Sections were then reacted with 3% H_2_O_2_ for 5 min, washed with TBS, incubated with TdT equilibration buffer for 20 min, followed by the incubation with TdT labeling reaction mixture for 1.5 h at 37°C in a humidified chamber. After TBS washing, the reaction was terminated by stop buffer solution for 5 min and washed and then blocked with blocking buffer for 10 min, followed by immersing the slides in conjugate solution for 30 min in a humidified chamber. After TBS washing, the sections were incubated with DAB solution for 15 min and rinsed with dH_2_O, followed by the examination under the light microscope. The samples were performed blindly to the animal treatment groups.

### Histopathological study

To determine any morphological changes, a kidney was removed and cut in a half along the transverse axis, fixed in 10% neutral buffered formalin and embedded in paraffin. Paraffin-embedded specimens were cut into 5-μm-thick sections, mounted on microscope slides and stained with Haematoxylin and Eosin for general histological assessment. The samples were examined under a light microscope for tubular and glomerular changes by an observer blinded to the animal treatment groups.

### Statistical analysis

The data were analyzed using SPSS17.0 statistical software (Chicago, Ill., USA). All data were expressed as the mean ± SEM. For the comparison between multiple treatments, a one-way ANOVA followed by Fisher's Least significant difference test (LSD) was used. P < 0.05 was considered statistically significant.

## Results

### The effects of atorvastatin on renal function in gentamicin-treated rats

**Fig 1:**
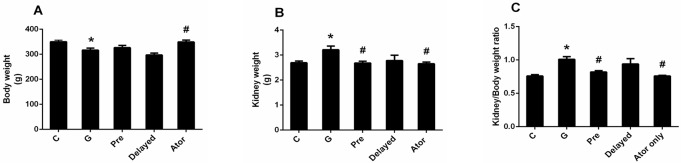
Effects of atorvastatin on the body weight (BW) (A); kidney weight (KW) (B); and KW/BW ratio (C). Bar graph indicates mean ± SEM. (n = 6 rats in each group). *P < 0.05 compared to the control group. ^#^P < 0.05 compared to the gentamicin-treated group. C: control group; G: gentamicin-treated group; Pre: atorvastatin pretreatment group; Delayed: delayed treatment group and Ator: atorvastatin group.

**Fig 2:**
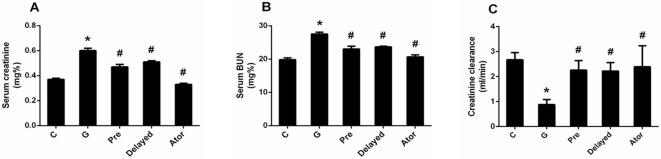
Effects of atorvastatin on serum creatinine (A); serum BUN (B); and creatinine clearance (C). Bar graph indicates mean ± SEM. (n = 6 rats in each group). *P < 0.05 compared to the control group. ^#^P < 0.05 compared to the gentamicin-treated group. C: control group; G: gentamicin-treated group; Pre: atorvastatin pretreatment group; Delayed: delayed treatment group and Ator: atorvastatin group.

In this study, there was no animal died before the endpoint of experiment. Gentamicin treatment caused a significant decrease in body weight (P < 0.05) when compared with that of the control and atorvastatin groups (Fig 1A). Kidney hypertrophy as indicated by the significant increase in kidney weight and kidney/body weight ratio was observed in gentamicin-treated group (P < 0.05) (Fig 1B and 1C) when compared with the control or atorvastatin group. In addition, the apparent increase in serum BUN and creatinine levels and a significant decrease in creatinine clearance in gentamicin-treated group compared with the control or atorvastatin groups (P < 0.05) (Fig 2A, 2B and 2C) indicated that renal function impairment was induced by gentamicin treatment. Kidney hypertrophy was significantly attenuated in atorvastatin pretreatment as compared to gentamicin-treated rats (P < 0.05) (Fig 1B and 1C). Atorvastatin could not only prevent kidney dysfunction but also reverse renal impairment, leading to an improvement of renal function when compared with the gentamicin-treated rats (Fig 2). However, only atorvastatin pretreatment could improve kidney hypertrophy. Atorvastatin treatment alone had no effect on renal function when compared with control.

### Effect of atorvastatin on gentamicin-induced renal inflammation

**Fig 3:**
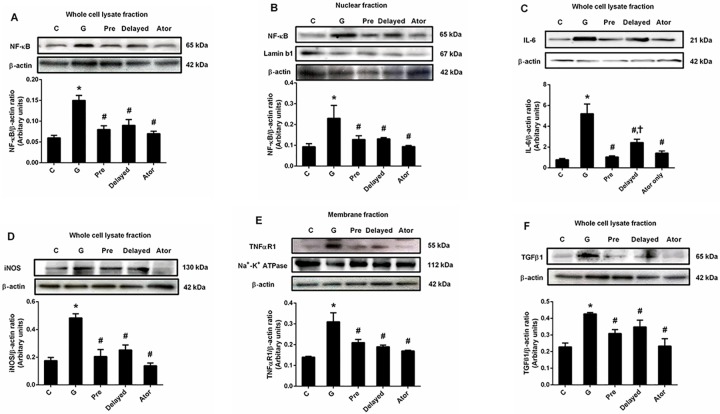
Effects of atorvastatin on the expression of NF-κB, IL-6, iNOS, TNFαR1 and TGFβ1 in the renal cortical tissue. Immunoblot analysis for expressions of NF-κB in the whole cell lysate fraction (A); NF-κB in nuclear fraction (B); IL-6 in whole cell lysate fraction (C); iNOS in whole cell lysate fraction (D); TNFαR1 in membrane fraction (E) and TGFβ1 in whole cell lysate fraction (F) in renal cortical tissues normalized to ß-actin. Lamin b1 or Na^+^-K^+^ ATPase was used as a marker for the nuclear or membrane fraction, respectively. Bar graph indicates mean ± SEM. (n = 6 rats in each group). *P < 0.05 compared to the control group. ^#^P < 0.05 compared to the gentamicin-treated group. ^†^P < 0.05 compared to the pretreatment group. C: control group; G: gentamicin-treated group; Pre: atorvastatin pretreatment group; Delayed: delayed treatment group and Ator: atorvastatin group.

The expression of NF-κB in both whole cell lysate and nuclear fractions from renal cortical tissues was significantly higher in gentamicin-treated rats than those of the control and atorvastatin alone groups (P < 0.05) (Fig 3A and 3B). These results indicated the activation of NF-κB induced by gentamicin treatment. Moreover, the significant increases in the expressions of IL-6 and iNOS in whole cell lysate fraction and TNFαR1 in the membrane fraction from renal cortical tissues were observed in the gentamicin-treated group compared to those from the control and atorvastatin alone groups (P < 0.05) (Fig 3C, 3D and 3E). This study demonstrated that the expressions of NF-κB, IL-6, iNOS and TNFαR1 were markedly decreased by atorvastatin pretreatment (P < 0.05). The treatment with atorvastatin on day 10 to 15 of experiment could also reduce the inflammation induced by gentamicin (P < 0.05) although IL-6 was still markedly elevated when compared with atorvastatin pretreatment group. In addition, it has been reported that infiltration of macrophage accompanied with myofibroblasts, TGFβ and endothelin might contribute to the development of renal fibrosis in gentamicin-treated rat [26]. The expression of TGFβ1, a key mediator of renal fibrosis, in renal cortical tissues [27], was significantly increased in the gentamicin group when compared to that of the control and atorvastatin groups (P < 0.05) (Fig 3F). Atorvastatin pretreatment and delayed treatment shared similar efficacy in attenuating TGFβ1 expression, leading to renal fibrosis reduction compared to that seen in the gentamicin-treated rats (P < 0.05). Atorvastatin treatment alone had no effect on NF-κB, iNOS, TNFαR1 and TGFβ1 expressions when compared with control. These findings could indicate that atorvastatin treatment attenuated renal inflammation and fibrosis induced by gentamicin.

### The effects of atorvastatin on renal ER stress

**Fig 4:**
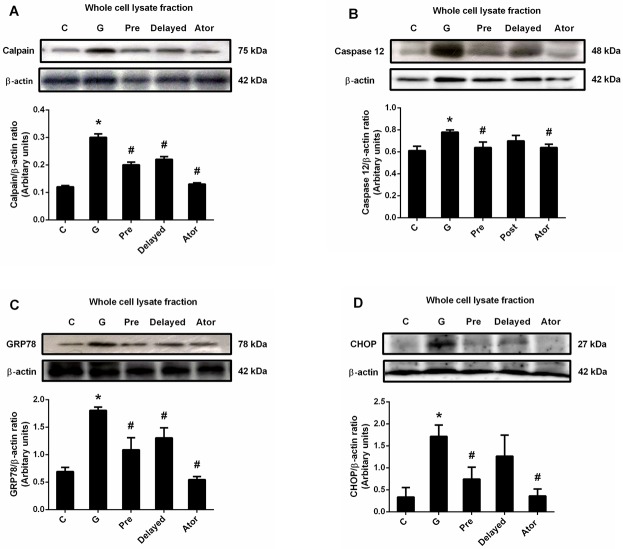
Effects of atorvastatin on the expression of calpain, caspase 12, GRP78 and CHOP. Immunoblot analysis for calpain (A); caspase 12 (B); GRP78 (C) and CHOP (D) expressions in the whole cell lysate fraction of renal cortical tissues normalized to ß-actin. Bar graph indicates mean ± SEM. (n = 6 rats in each group). *P < 0.05 compared to the control group. ^#^P < 0.05 compared to the gentamicin-treated group. C: control group; G: gentamicin-treated group; Pre: atorvastatin pretreatment group; Delayed: delayed treatment group and Ator: atorvastatin group.

To investigate the effect of atorvastatin on the ER stress-mediated cell death signaling pathway in gentamicin-induced nephrotoxicity, the ER stress-related protein expression was determined. Gentamicin treatment caused significant increases in calpain, caspase 12, GRP78 and CHOP protein expressions (P < 0.05), indexes of ER stress markers, in renal cortical tissue when compared to both the control and the atorvastatin groups (Fig 4). Atorvastatin pretreatment abolished the increases in calpain, caspase 12, GRP78 and CHOP expressions induced by gentamicin treatment (P < 0.05). However, only the significant decreases in calpain and GRP78 but not caspase 12 and CHOP expressions were observed in atorvastatin delayed treatment (P < 0.05) when compared with those of the gentamicin-treated group. Atorvastatin treatment alone had no effect on ER stress when compared with control. These results indicated that atorvastation treatment could inhibit ER stress pathway induced by gentamicin treatment.

### The effects of atorvastatin on renal apoptosis

**Fig 5:**
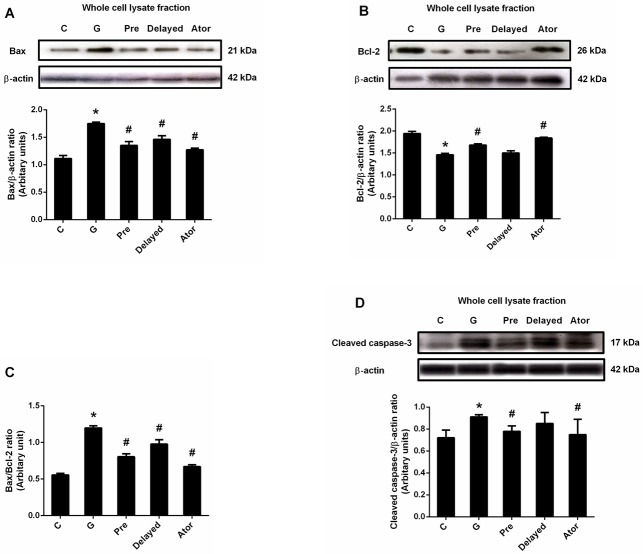
Effects of atorvastatin on the expression of Bax, Bcl-2 and cleaved caspase-3. Immunoblot analysis for Bax (A) Bcl-2 (B) Bax/ Bcl-2 ratio (C) and cleaved caspase-3 (D) expressions in the whole cell lysate fraction of renal cortical tissues normalized to β-actin. Bar graph indicates mean ± SEM. (n = 6 rats in each group). *P < 0.05 compared to the control group. ^#^P < 0.05 compared to the gentamicin-treated group. C: control group; G: gentamicin-treated group; Pre: atorvastatin pretreatment group; Delayed: delayed treatment group and Ator: atorvastatin group.

**Fig 6:**
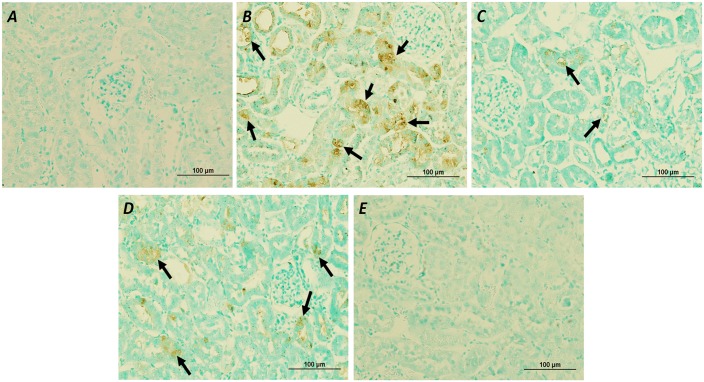
Effects of atorvastatin on apoptotic cells in kidney tissues. Apoptotic cells within kidney tissues were evaluated by TUNEL assays in control (A), gentamicin-treated (B), atorvastatin pretreatment (C), atorvastatin delayed treatment (D) and atorvastatin only (E) rats. TUNEL-positive cells were predominantly located at the renal tubules of the renal cortex (black arrow).

To elucidate the effect of atorvastatin on gentamicin-induced renal cell apoptosis, the apoptosis related pro-apoptotic and anti-apoptotic protein expressions and TUNEL assay were examined. Significant enhanced Bax and cleaved caspase-3 expressions and marked reduced Bcl-2 expression in renal cortical tissue were observed (P < 0.05) in the gentamicin group compared to those of the control and atorvastatin groups (Fig 5A, 5B and 5D). The Bax/Bcl-2 ratio was also significantly increased in gentamicin-treated rat (P < 0.05) (Fig 5C). The results showed that the numbers of TUNEL-positive cells were observed in gentamicin-treated rats, predominantly located at the renal tubules of the renal cortex when compared with the control group (Fig 6). Atorvastatin pretreatment significantly reduced the expressions of the Bax, cleaved caspase-3 and Bax/Bcl-2 ratio and apparently increased Bcl-2 expression when compared to the gentamicin group (P< 0.05). The enhanced expressions of Bax and Bax/Bcl-2 ratio in gentamicin-treated rats were significantly reversed with the delayed treatment with atorvastatin (P <0.05). However, delayed treatment with atorvastatin could not down-regulate the expression of cleaved caspase-3 or enhance Bcl-2 expression. Atorvastatin pre and delayed treatments could reduce TUNEL-positive cells as compared to the gentamicin-treated group. Atorvastatin treatment alone had no effect on Bax, Bcl-2 and cleaved caspase-3 expressions when compared with control. These results suggest that atorvastatin treatment attenuates gentamicin-induced apoptosis in rat renal tubular cells by inhibiting ER stress and apoptosis pathway.

### The effects of atorvastatin on renal histology

**Fig 7:**
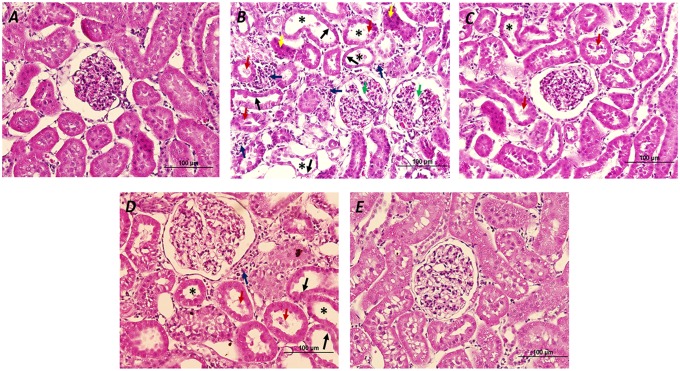
Effects of atorvastatin on histological changes of the kidneys. Hematoxylin- and Eosin-stained kidney tissue sections were performed in control (A); gentamicin-treated (B); atorvastatin pretreatment (C); atorvastatin delayed treatment (D) and atorvastatin only (E) groups. Glomerular degeneration (green arrow), mononuclear cells infiltration (blue arrow), pyknotic nuclei (yellow arrow), renal tubular desquamation (red arrow), renal tubular atrophy (black arrow) and renal tubular dilatation (asterisk) were indicated.

The results showed that gentamicin-treated rats demonstrated glomerular damage, tubular atrophy, tubular dilatation, cellular desquamation, pyknotic nuclei and interstitial mononuclear cells infiltration. Atorvastatin pre and delayed treatments could preserve kidney morphology in the levels of glomerular, tubular and interstitial cells in this study (Fig 7).

## Discussion

Gentamicin, an aminoglycoside antibiotic, is the most clinically used due to its wide spectrum of activities against gram-negative bacterial infections [28]. However, there are limitations to its use due to nephrotoxic side effects. 10–20% of all cases of acute renal failure are due to these nephrotoxicity of this antibiotic. Some clinicians suggest a daily dose of 4 to 7 mg/kg once daily for all patients with normal renal function. In this study, we would like to induce renal dysfunction and use atorvastatin treatment to reverse this impairment. Thus, we selected the supramaximal dosage or 100 mg/kg/day of gentamicin in order to induced nephrotoxicity according to the previous studies [23,24,29]. In addition, the preliminary data, rats receiving gentamicin 100 mg/kg/day for 15 days via intraperitoneal injection demonstrated renal dysfunction as shown by the increased serum creatinine and BUN.

Numbers of evidence showing that statins exert beneficial effects independent of its lipid lowering ability, called pleiotropic effects [30]. Clinical studies showed that atorvastatin, a 3-hydroxy-3-methylglutaryl coenzyme A (HMG-CoA) reductase inhibitor, administration improved ventricular ejection fraction, attenuated adverse effect ventricular remodeling and depressed the inflammation process in heart failure patients [31,32]. Furthermore, atorvastatin also decreased the apoptosis of myocardial cells in rat heart failure model [33]. Nevertheless, the role of atorvastatin in gentamicin-induced nephrotoxicity is still poorly known.

Gentamicin cytotoxicity occurs in the cell types in which the drug accumulates. In the kidneys, these cells constitute the tubular epithelial cells in the cortex, mainly in the proximal tubule of experimental animals and humans [34,35]. The expression of a transporter of proteins and cations, the giant endocytic complex formed by megalin and cubilin, which is restricted to the proximal tubule, is consistent with a higher accumulation of gentamicin and responsible for the transport of gentamicin into these cells. We therefore investigated protein expression in the kidney cortex where the proximal tubule is located. The present study showed that nephrotoxicity induced by gentamicin was characterized by a marked increase in serum creatinine and BUN levels along with a significant reduction in rate of creatinine clearance and histological changes. Moreover, kidney injury in gentamicin-treated rats was evidenced by the increased renal expressions of NF-κB as well as IL-6, iNOS and TNFαR1. These effects were accompanied by increases in the ER stress markers (including calpain, caspase 12, GRP78 and CHOP) and pro-apoptotic Bax and cleaved caspase-3 with a decrease in antiapoptotic Bcl-2 which induced renal apoptosis. Atorvastatin treatment at dose 10 mg/kg significantly improved renal function, and ameliorated renal inflammation, ER stress and apoptosis in gentamicin-treated rat.

Renal injury as a consequence of gentamicin-induced tubular necrosis has been shown to accompany with increased inflammatory events at the site of injury and to enhance the migration of monocytes and macrophages to the site of tissue damage [4]. NF-κB is a key transcription factor in the renal inflammatory process by regulating the gene expression of cytokines, chemokines, adhesion molecules and iNOS which provoke kidney damage [36–38]. We determined the classical pathway of NF-κB which activated by inflammation or ROS induced by gentamicin injection. In the classical pathway, NF-κB proteins are bound and inhibited by IκB proteins. Proinflammatory cytokines including LPS, growth factors, and antigen receptors activate an IKK complex then IκB proteins are phosphorylated. Phosphorylation of IκB leads to its ubiquitination and proteasomal degradation resulting in freeing IκB complexes. Active NF-κB translocates to the nucleus inducing target gene involve proinflammatory cytokine [23,36,39,40]. In this study, gentamicin induced renal injury and activated inflammatory response, free NF-κB dimers translocate to nucleus and activate the target genes such as iNOS and IL-6 expressions. The increased activation and nuclear translocation of the NF-κB were evidenced by the increased NF-κB expression in both whole cell lysate and nuclear fractions in renal cortical tissue. The activation of NF-κB was accompanied with the increased iNOS and TNFαR1 expressions and interstitial mononuclear cells infiltration in gentamicin-treated rats suggesting that NF-κB may play a role in renal inflammation in this study. Previous study in type 2 diabetic rat showed that the endothelial dysfunction was resulted from overexpression of TNFαR [41]. Thus, an increased membrane expression of TNFαR1 in the present study might imply an elevation of TNFα activity in the kidneys in gentamicin-treated rat. The results of this study corroborated those found in a previous study which demonstrated that an increased NF-κB activation in gentamicin-treated rats was followed by increasing the synthesis of inflammatory substances. Moreover, TNFα also activates the NF-κB pathway, thus resulting in amplification of the inflammatory response [42]. Atorvastatin pretreatment or delayed treatment decreased the expressions of NF-κB, IL-6, iNOS and TNFαR1 and interstitial mononuclear cells infiltration in gentamicin-treated rat. These findings suggest that atorvastatin improves renal inflammation by attenuating the activation of the NF-κB pathway. This is in agreement with the anti-inflammatory effects of atorvastatin found in heart failure [43], obstructive uropathy [44] and endotoxemia [45]. Additionally, previous studies demonstrated that gentamicin increased macrophage infiltration and elevated TGFβ1 level leading to the progression of tubulointerstitial nephritis [26,46]. An expression of TGFβ1 showed a strong correlation with fibrosis of smooth muscle layer in rats with unilateral ureteral obstruction [44]. In animal models with ureter obstruction, statins have been reported to inhibit inflammatory mediators and macrophage infiltration [47], and to suppress TGFβ1 expression and extracellular matrix production [48,49]. The current results, compatible with the study of Chuang et al [44], showed that atorvastatin pre or delayed treatment markedly down regulated not only IL-6, iNOS and TNFαR1 but also TGFβ1 expression in gentamicin-treated rats. The reduction in inflammatory gene expression is in accordance with the reduced inflammatory infiltrate seen in the histological study in atorvastatin treatment and previous study [15]. These imply that atorvastatin pre or delayed treatment may ameliorate the tissue damage in gentamicin-induced nephrotoxicity via the inhibition of the TGFβ1 expression and by suppression of pro-inflammation cytokines production.

The significant increases of calpain, caspase 12, GRP78 and CHOP expressions in renal cortical tissue in this study reflected that gentamicin treatment induced ER stress and caused the activation of ER-mediated cell death markers. The activation of ER stress is one of the underlying mechanisms enabling the protection and repair in stress-induced cellular disorder by inducing cell apoptosis [33]. Calpain is responsible for cleaving pro-caspase 12 to active caspase 12. In this study, an up-regulation of cleaved caspase 3 in gentamicin-treated rats showed a correlation with an increased expression of caspase 12. Caspase 12 was translocated from ER to the cytosol and procaspase 9 was directly cleaved, which, in turn, activated caspase 3 leading to cell death [50]. It was noteworthy that the increased expression of calpain, caspase 12, GRP78 and CHOP were significantly suppressed by atorvastatin treatment, particularly pretreatment. Consistently, atorvastatin had been shown to decrease the rate of cell apoptosis and the expression of proteins involved in the ER stress response and apoptosis such as GRP78, caspase 12 and C/EBP homologous protein in myocardial cells in a rat model with post-myocardial infarction heart failure [33]. In this study, gentamicin treatment also caused a reduced expression of Bcl-2 accompanied with the elevated expression of Bax, the Bax/Bcl-2 ratio, cleaved caspase 3 and the increased TUNEL-positive cells, suggesting that gentamicin-induced renal damage was associated with the activation of apoptotic pathway. Atorvastatin pre or delayed treatment provided renoprotection by inhibiting Bax overexpression induced by gentamicin with an enhanced Bcl-2 expression, although the decreased expression of cleaved caspase-3 was presented only in pretreatment. According to the report that NF-κB activation promoted gentamicin-induced apoptosis in rat tubular cells [5], the anti-inflammatory effects of atorvastatin may partly be attributed to its suppression of gentamicin-induced apoptosis. In this study, it might be suggested that atorvastatin protects against renal tubular cell apoptosis induced by gentamicin partly by down-regulating the activation of ER stress and NF-κB pathways. These proposed mechanisms are confirmed by the previous study demonstrating that atorvastatin acts a potent scavenger of free radicals in the kidney leading to the inhibition of MAPK and NF-κB signaling pathways to prevent gentamicin-induced renal toxicity [20].

In conclusion, this study provides evidence that atorvastatin can reduce gentamicin induced NF-κB activation as well as inhibiting inflammatory, ER stress and apoptotic pathways. The effect of atorvastatin in protecting from renal damage induced by gentamicin seems to be more effective when it was given prior to gentamicin exposure.
